# Mortality of People with Parkinson’s Disease in a Large UK-Based Cohort Study: Time Trends and Relationship to Disease Duration

**DOI:** 10.1002/mds.28727

**Published:** 2021-08-05

**Authors:** Olaitan Okunoye, Laura Horsfall, Louise Marston, Kate Walters, Anette Schrag

**Affiliations:** 1 Department of Clinical and Movement Neurosciences, University College London, London, United Kingdom; 2 Department of Primary Care and Population Health, University College London, London, United Kingdom

**Keywords:** Parkinson’s disease, mortality, trends, disease progression, sociodemographic factors, primary care

## Abstract

**Background:**

Parkinson’s disease (PD) is associated with increased mortality, but little is known about changes over time, and relationship to disease progression.

**Objectives:**

To explore how PD mortality rates have changed over time and their relationship to disease duration and demographics using a large population-based cohort in the UK.

**Methods:**

We included individuals aged 50+ years with a first recording of PD diagnosis and at least two prescriptions of any antiparkinsonian drug actively registered within a general practice from 2006 to 2016 and up to six frequency-matched controls from The Health Improvement Network (THIN) database. We estimated adjusted mortality rates using multivariable Poisson regression.

**Results:**

A total of 10,104 people with a diagnosis of PD and 55,664 people without PD were included. Overall, PD was associated with slightly increased mortality compared to non-PD controls (adjusted mortality rate ratio: 1.14; 95% CI: 1.03 to 1.19). Adjusted mortality rates per 1000 person-years at risk for people with PD approximately doubled in the 5 years following diagnosis from 43 (95% CI: 38 to 48) to 75 (95% CI: 64 to 85). Following adjustments for age, gender, and time since diagnosis, mortality rates between 2007 and 2016 declined more slowly for people with PD (2% per year; 95% CI: 0%–4%) compared to people without PD (5% per year; 95% CI: 3%–6%).

**Conclusions:**

Whilst mortality in PD is only slightly increased overall, it gradually increases with advancing disease. There has been a decline in mortality in PD over time, but this decrease was less pronounced than that in the general population.

Despite ongoing progress in the treatment of Parkinson’s disease (PD), mortality is increased compared to the general population.^1–3^ However, previous population-based studies often used prevalent cohorts with varying disease duration rather than using more informative incident cohorts followed up from diagnosis, leading to possible overestimation of mortality.^4–10^ Other population-based mortality studies have included patients with progressive supranuclear palsy, corticobasal degeneration, and multiple system atrophy, which have higher mortality,^11^ or were recruited from a movement disorder clinic which carries the risk of raising selection bias.^12^

Age-adjusted mortality over the age of 50 years has been decreasing in the UK over the last decade,^13,14^ but there are no data on whether this is also the case for patients with PD. There have been considerable changes in the availability and use of symptomatic treatments for PD, including pharmacological, surgical, and non-pharmacological treatments, but at the present time there are no disease-modifying treatments available.^15,16^ Recent treatment changes are intended to improve the quality of life of patients, but it is unclear whether they have also affected life expectancy. We therefore examined whether mortality of people with PD over the time period 2006–2016 has changed in a similar pattern compared to that of the general population. Estimation of survival and mortality rates are possible through analysis of data in large primary care databases that contain information on diagnoses and death, such as The Health Improvement Network (THIN). The aim of this study was to estimate trends in mortality for people with PD in the UK in comparison with people without PD in this database, and to explore associations of mortality with disease duration and sociodemographic factors.

## Methods

Information from general practices in the UK contributing data to THIN during the time period January 2006–December 2016 were examined in this cohort study. We used data from practices that met the quality assurance criteria for acceptable levels of data recording^17^ and acceptable standards of mortality reporting.^18^ Acceptable Mortality Reporting (AMR) date is a measure of the quality of death recording in THIN. This is the year from which an individual general practice is considered to have mortality records which are proportional to that from the Office for National Statistics (ONS).^18,19^ Acceptable Computer Usage (ACU) date is a quality filter to determine when a general practice was using electronic recording fully; only practices which met these quality standards were included in this study.

### Data Source

The Health Improvement Network (THIN) is one of the largest longitudinal, primary care databases in the UK containing electronic medical records of more than 12 million patients (https://www.iqvia.com) and covering around 6% of the UK population.^20^ It is a rich source of continuous primary care data on patients’ consultations and prescribing in the UK. Information for the database is generated by collecting anonymized patient data from more than 700 participating general practices. These are generally representative of UK general practices with regard to age, gender, geographical location, and practice size.^21^ Medical diagnoses and patient symptoms are recorded by general practitioners (GPs) using Read codes, a hierarchical coding system used for recording patients’ clinical summary. For each individual registered with a general practice, information on age, gender, symptoms, medical diagnosis, prescriptions, and referrals to secondary care are recorded. Linked data on local area deprivation in Townsend quintiles (measure of social deprivation with Townsend quintiles from 1 [most affluent] to 5 [most deprived]) is also available, linked via the postal (zip) code to UK 2011 Census data. This is generated from a combination of measures which take into account an individual’s occupation, car possession, overcrowded housing, and unemployment.^22^ In addition, information on blood pressure, smoking status, height, weight, and laboratory test results are all recorded in THIN. Information on death is also available in THIN. All data are de-identified, processed, and validated by CSD Medical Research UK (https://www.iqvia.com).

### Study Design

The study population included adults who were 50 + years of age and had been actively registered with a general practice for at least 6 months during the time period 1 January 2006 to 31 December 2016 (n = 3,195,391). The earliest date of PD diagnosis Read code recording or antiparkinsonian drug code recording was taken as the index date. Participants entered the cohort if they met the inclusion criteria or on the randomly matched date (index date) for individuals in the comparator group (*Non-PD Cohort*) and were followed up until they died (n = 11,198), de-registered with the GP practice (n = 14,722), or the GP practice stopped contributing data to THIN (n = 37,464), whichever was earliest. In 2003, the National Health Service South-East Multicentre Research Ethics Committee gave approval for the use of the THIN database. This study was approved by IQVIA Medical Research Scientific Review Committee in June 2019 (SRC Reference No. 19THIN034).

### Inclusion and Exclusion Criteria

We excluded all people with a history of PD (prior to study entry) and those with restless leg syndrome without PD (n = 31) who were treated with dopamine agonists. In addition, individuals with diagnosis in the first 6 months after registration with a practice were excluded (n = 3756) because they could represent a recording of medical information which may be retrospective rather than a true new recording of PD.^23^ For the analysis on time trends and disease duration, we started the study period 1 year from the index date only for those entering before and during 2007 to avoid follow-up time being too short for deaths to occur (Fig. S1).

The “*PD Cohort*” consisted of all adults aged 50 + years with first ever diagnosis Read code for PD and at least two prescriptions of any of five major classes of antiparkinsonian medications (levodopa-containing medications, dopamine receptor agonists, amantadine, monoamine oxidase B inhibitors [rasagiline and selegiline], and catechol-O-methyltransferase inhibitors [entacapone and tolcapone] during the time period January 2006–December 2016. This case definition has been shown to have a validity of 90% in the General Practice Research Database (GPRD),^24^ in which there is an overlap with THIN in about 60% of patients.^25^ This same case definition has been used in a previous study.^26^ Read code lists to identify electronic recording of PD diagnosis and drug code lists to identify the five major classes of antiparkinsonian medications were created using published guidelines.^27^

The “*General Population (Non-PD Cohort)*” comprised a frequency-matched random sample of up to six people with no record of PD. The non-PD cohort was frequency-matched within each practice on age, gender, and calendar year using a randomly assigned index date.

### Outcome

The main outcome was all-cause mortality. We identified individuals who died during the follow-up through their death records in THIN.

### Statistical Analysis

For descriptive purposes, we categorized mortality by calendar year, social deprivation, and 10-year age-bands: 50–59, 60–69, 70–79, 80–89, and 90+ years.

A multivariable Poisson model was used to estimate the adjusted mortality rates. Age was used as the time-scale and data on age and calendar year were split by 1-year intervals. We estimated unadjusted and mutually adjusted mortality rates and rate ratios for age, gender, calendar year, time since diagnosis or index date, smoking status, and social deprivation by PD status. We tested for multiplicative interactions between PD status and each of these variables. In order to calculate the mortality rate ratios and the marginal effects (adjusted mortality rates) adjusted for age group and other covariates, we used multivariable Poisson regression analyses. Whilst rate ratios have a baseline comparator, marginal effect on the other hand is a measurement of how much the mortality rate is predicted to vary per unit change in an exposure variable. Marginal effects for fixed values of calendar year were estimated while holding all other parameters at their observed values in the model and applying the delta method for estimation of the standard errors. We explored nonlinear relationships of mortality with age and calendar year using restricted cubic spline interpolation. We compared linear models with different spline transformations (3–5 knots) and used the Akaike’s Information Criterion (AIC) and Bayesian Information Criterion (BIC) to check whether additional knots or cubic spline transformations improved the model fit while avoiding unneeded complexity. The *P* values for categorical variables and multiplicative interaction terms were estimated by using Wald tests. We included general practice identifiers and calculated robust standard errors in order to account for the effects of clustering of observations within GP practices. Negative binomial models were run, and the outputs were compared. This allowed us to investigate overdispersion in the analyses.

All statistical analyses were conducted using Stata version 16MP (Stata Corporation, College Station, TX).

## Results

### Demographic Features

Within our study period of 1 January 2006 and 31 December 2016, 704 general practices met the criteria for acceptable mortality and data recording. We identified a total of 10,104 people with a diagnosis of PD (PD-cohort) with active enrolment in a general practice between the beginning 2006 and the end of 2016. There were more males in our PD cohort (60.7%). PD patients were matched with for age, gender, calendar year, and GP practice with 55,664 people without PD (non-PD cohort). The PD cohort had a lower percentage of people in the most deprived quintile (8.9% vs. 10.0%) and a lower percentage of people smoking (6.8% vs. 13.1%). In the PD cohort there were 2031 deaths while the non-PD cohort had 9167 deaths (*P* < 0.001; Table 1).

### Mortality Rates

The overall unadjusted mortality rate in people with PD (PD-cohort) was 56.06 per 1000 person-years (95% CI: 53.68–58.56) and that in the non-PD cohort was 50.07 per 1000 person years (95% CI: 49.05–51.10). After adjustment for age, gender, calendar year, social deprivation, and smoking, overall mortality rate was elevated in the PD-cohort compared to the non-PD cohort (mortality rate ratio 1.14, 95% CI: 1.09–1.20) (Table 2).

### Trends in Mortality Rates Over Time

**Fig. 1:**
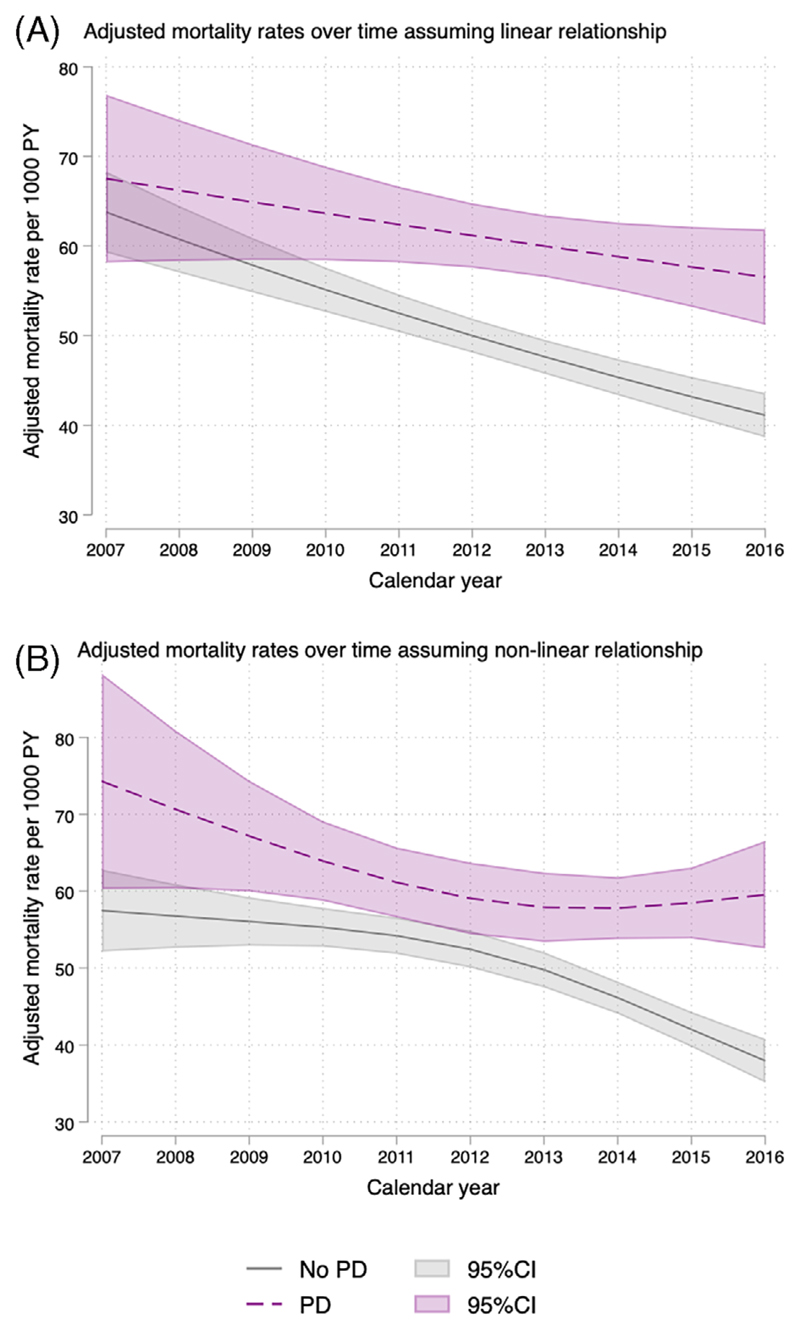
Mortality over time (A) on the linear scale adjusted for age, gender, social deprivation, and smoking; (B) on the nonlinear scale adjusted for age, gender, social deprivation, and smoking. PY, person-years; PD, Parkinson’s disease; CI, confidence interval. [Color figure can be viewed at wileyonlinelibrary.com]

The BIC favored a linear function for age whereas AIC favored a three-knot spline transformation and we have therefore reported both (Fig. 1). There was an interaction between PD status and calendar year (*P* value for interaction in spline transformed model = 0.0005). Adjusted mortality rates in the PD group declined over time by around 2% per year or 1.2 per 1000 person-years. However, adjusted mortality rates in the non-PD group decreased more dramatically at 5% per year or 2.4 per 1000 person-years over the time period observed. Further adjustment for smoking status and social deprivation had no meaningful impact on the estimates.

**Fig. 2:**
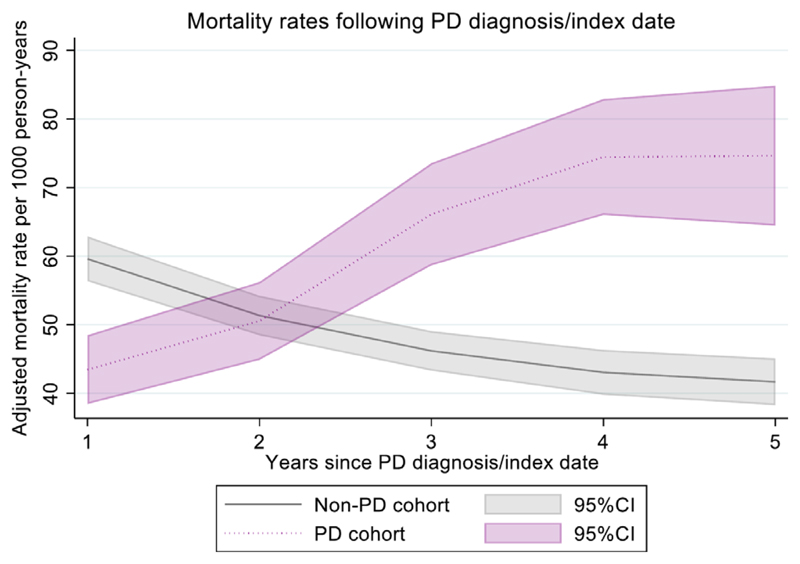
Mortality rates following Parkinson’s disease diagnosis/index date adjusted for age, gender, calendar year, social deprivation, and smoking. PD, Parkinson’s disease; CI, confidence interval. [Color figure can be viewed at wileyonlinelibrary.com]

There were also strong interactions between PD status and time since diagnosis/index date (*P* value for interaction <0.0001). Adjusted mortality rates per 1000 person-years were lower in people with PD than the general population in the year following diagnosis (Fig. 2), but gradually increased with each year from 43 (95% CI: 38–48) in the first year to 75 (95% CI: 64–85) at 5 years after diagnosis (Fig. 2 and Table S2). Conversely, adjusted mortality rates were decreasing in the non-PD cohort during their follow-up from index date, resulting in a gradually increasing mortality rate ratio with increasing years after diagnosis (*P* < 0.001; Fig. 2 and Table S2). We further examined time trends separately by year since diagnosis/index date up to 4 years since diagnosis/index date. There was no strong evidence of nonlinearity for calendar year within years since diagnosis/index date, and due to low number of events we only explored up to year 4. In the first year, overall mortality rates were lower in people with PD and declined at a similar rate to those without PD. However, we found that a differential decline in mortality rates was seen in the more recent years of data collection for PD patients more than 2 years into their diagnosis (Fig. S2).

In order to account for immortal time bias, we conducted a sensitivity analysis where start of follow-up was moved to the latest date of PD diagnosis using Read code recording or antiparkinsonian drug code.^28,29^ This showed no meaningful change in the results (Fig. S3).

### Other Factors Associated with Increased Mortality Rates

Adjusted mortality rates increased with age at a broadly similar rate for people with and without PD (Table 3 and Fig. S4) (*P* value for interaction in spline transformed model = 0.89). Men in both groups had similarly higher adjusted mortality rates compared to women (*P* value for interaction = 0.45) (Table 3). Adjusted mortality rates increased similarly with social deprivation in both groups and smoking status was also associated with increased mortality in both groups. There was some evidence that the differences in mortality rates for people with and without PD were more pronounced in the least deprived areas and in non/former smokers; however, the differences were small (Table 3).

In order to examine whether the validated but stringent PD inclusion criteria introduced selection bias and limited the generalizability of the findings, we also examined the differences in demographic characteristics when loosening the inclusion criteria from PD code plus two prescriptions to PD code plus one prescription and PD diagnosis code only. The sociodemographic characteristics were broadly similar across included/excluded groups although the more stringent criteria seemed to include more younger people and men (Table S3).

## Discussion

In this large, population-based study of more than 10,000 patients with PD and more than 50,000 in the control group without PD, the mortality rate ratio in the PD cohort was 1.14 times that of the general population indicating a modest overall increase in mortality associated with the condition. The mortality rate ratio in this study is within the range reported in the literature, but at the lower end of the previous published mortality results. In previous studies mortality ratios in people with PD have been reported to range from 0.9 to 3.8.^1^ Whilst in the lower range it is comparable to recent incident clinical and community-based cohort studies which have also reported similar mortality ratios.^1,30,31^ Previous register-based or population-based studies^4,7,8,10,32–35^ with higher rate ratios have included both incident and prevalent cases,^7^ while others included prevalent PD cases which are likely to have higher mortality than newly diagnosed PD cases. These cases are likely to have longer disease duration and severe disease resulting in higher mortality.^4,8,10,32,33,35,36^ Other population-based studies^31,37–39^ on survival of incident patients with PD are limited by small number of cases^31,36,39^ and often also included people with other causes of parkinsonism such as multiple system atrophy, corticobasal degeneration, or progressive supranuclear palsy which have been reported to have higher mortality.^11^

Our findings suggested that men with PD had a slightly higher mortality over women with PD, but the difference was small and not different to the control group without PD. Previously, a Sydney multicentre study showed no differences in the sexes^40^ but other studies have reported increased mortality in men with PD compared to women with PD^4,41^ There are speculations that female sex hormones (estrogens) have some neuroprotective effect on neuronal cell death,^42^ and reports of animal studies showed that the potential beneficial effect of estrogens is probably due to their anti-oxidant effects.^43^ Other available data are conflicting^43–46^ and further large observational studies are proposed. We also found no strong differences in mortality between people with and without PD as regards influence of lower socioeconomic status and smoking in this study, both of which are associated with increased mortality.^47^

In comparison to the general population, mortality rates were lower in patients with PD than the general population in the year following diagnosis, but gradually increased in patients with PD with longer disease duration, with the excess mortality of people with PD continuing to increase with each year after the diagnosis of PD. It is possible that patients who present with symptoms leading to diagnosis of PD receive greater medical attention and more investigations at the time of diagnosis, although referral bias due to patients with short life expectancy not being referred cannot be excluded. However, increasing disease progression with advancing disease was associated with higher mortality. This has been reported in a recent systematic review on mortality in PD in which rate ratios are reported to increase with disease duration.^1^ In the Rotterdam study, mortality was reported to increase with disease duration with ratios increasing by 1.03 (95% CI: 0.99–1.07) per year.^7^ Another study from the USA, however, reported increased mortality risk across all categories of disease duration including disease duration below 2 years (rate ratio 2.0, 95% CI: 1.03–3.88).^48^ The reason for this increasing mortality rate in PD with longer disease duration may be comorbidities, increasing disease severity, or complications of PD. These will need to be identified and addressed in order to develop interventions to reduce mortality in PD.^41^

During our follow-up period, adjusted mortality rates per 1000 person-years in people with PD declined slightly from 69 (95% CI: 60–79) in 2007 to 59 (95% CI: 53–64) in 2016. This is in keeping with previous studies in the UK, which showed a steady slight decline in mortality in PD between 1993 and 2006^49^ as well as earlier periods.^50,51^ Conversely, recent research from Northern Italy^52^ showed an increasing trend in mortality of people with PD from 1.9% in 2008 to 2.4% in 2015. This study was based on a shorter interval and was limited by mortality records as the authors included other types of parkinsonism (with higher mortality) in their analysis and reported that they could not ascertain the specificity of PD in death certificates leading to possible overestimation of PD mortality. Differences in healthcare systems in different countries could also have contributed to differences in PD mortality.

However, in our study mortality rates per 1000 person-years at risk in the general population fell from 63 (95% CI: 58–67) in 2007 to 41 (95% CI: 39–44) in 2016 over time in keeping with the previously reported declining mortality rates during that decade. Reports from ONS^13^ and Public Health England^14^ showed a significant decline in age-specific mortality rates in both men and women aged 50+ years in the UK population between 2006 and 2016. The explanation for this reduction in mortality was attributed to falling rates from cardiovascular diseases. As there was a slower decline in mortality rate in the PD group, this resulted in an increasing mortality interval between patients with PD and the control population over that time period. These data suggest that improvement in mortality in the age group 50+ years in the general population is not seen in the same way in people with PD. It is possible that interventions leading to lower cardiovascular death rates are either not implemented or as effective to the same degree in patients with PD. Alternatively, the process of PD itself is the primary determinant of mortality, particularly in advancing disease, and therefore overshadows improvements in cardiovascular or other health. This interpretation is supported by our subanalysis of mortality within fixed time periods, which suggested that the increase in the mortality gap is particularly seen with longer disease duration. There are very few comparable studies. In a previous study in Ontario, Canada from 1996 to 2013 the mortality in people with PD declined by 5.5% over 18 years.^53^ Whilst this study did not include a control group, precluding a direct comparison with mortality in the general population, the authors reported that mortality in the general population had previously been found to have decreased by 19% during a similar time period.^53^

### Strength and Limitations

The main strength of this study is the prospective design with a follow-up of 10 years using data from a large primary care database (THIN) which is generalizable to people with PD living in the UK. This database has provided information on mortality of the PD cohort and the comparator non-PD cohort which were derived from the UK general population. Another strength of our study is that we evaluated mortality in newly diagnosed cases (incident cohort) of PD rather than prevalent cases which may lead to overestimation of rate ratios. It is reported that use of prevalent cases may cause higher estimates of rate ratios.^36^ This suggests that our relatively low mortality rates represent a better estimate for the overall population of patients with PD. In addition, a range of possible demographic and health confounders that could distort the results were taken into consideration while developing our robust statistical approach for analyzing mortality data in this cohort study.

There are, however, limitations with respect to analysis of routine healthcare data. We cannot rule out underdiagnosis of PD as registration in the database requires health-seeking behavior as well as diagnosis and those who do not attend a GP practice may be missed. Another limitation of this study is that we did not examine these patients but relied on GP codes for PD for clinical diagnoses which may result in mis-classification of PD cases. However, the validity of significant diagnoses in primary care databases is high,^54,55^ including for PD,^24^ and in most instances this will be recorded following a diagnosis made in secondary care. In any observational study using routine data the potential for residual confounding cannot be ruled out. In particular we were unable to include factors such as ethnicity or some social factors (eg, having a carer, marital status) due to the high number of missing data. Nevertheless, adjustment for smoking and socioeconomic status as confounders in addition to age- and gender-standardized mortality rate ratios increased the applicability of the results from this study.

## Conclusions

Our large population-based cohort study found that mortality rates in PD in the UK between 2006 and 2016 were slightly increased compared to the general population but mortality trends across gender, age, smoking status, and social deprivation were broadly similar to people without PD. Mortality was lower than in the general population in the year of diagnosis but increased year on year with longer disease in people with PD. Whilst overall mortality rates slightly declined in the PD group over the 10-year time period studied, mortality rates in people without PD declined at a faster rate leading to a relatively large differential in the most recent year of data (2016). These data suggest that progression of PD is associated with increasing mortality and that a decrease in mortality will require treatments to address the underlying disease progression, complications, or associated comorbidities.

## Supplementary Material

Supplementary information

## Figures and Tables

**Table 1:** Baseline characteristics for Parkinson’s disease (PD) and non-PD cohort

Variable	PD cohort (n = 10,104)	Non-PD cohort (n = 55,664)	^a^*P* value
Died, n	2031	9167	<0.001
Gender, n (%)			
Men	6135 (60.72)	33,778 (60.68)	0.945
Women	3969 (39.28)	21,886 (39.32)	
Age group (y), n (%)			
50–59	790 (7.82)	4403 (7.91)	0.705
60–69	2328 (23.04)	12,961 (23.29)	
70–79	4187 (41.46)	23,172 (41.63)	
80–89	2554 (25.28)	13,941 (25.05)	
>90	245 (2.40)	1187 (2.31)	
Townsend score			
1 (least deprived)	2811 (27.82)	13,746 (24.69)	<0.001
2	2320 (22.96)	12,399 (22.27)	
3	1901 (18.81)	10,815 (19.43)	
4	1441 (14.26)	8728 (15.68)	
5 (most deprived)	895 (8.86)	5565 (10.00)	
Missing data	736 (7.28)	4411 (7.92)	
Smoking status			
Non-smoker	5500 (54.43)	24,501 (44.02)	<0.001
Ex-smoker	3126 (30.94)	18,624 (33.46)	
Current smoker	690 (6.83)	7280 (13.08)	
Missing data	788 (7.80)	5259 (9.45)	

Abbreviations: PD, Parkinson’s disease; y, years.

a Chi-squared test.

**Table 2:** Overall mortality rates and ratios in the Parkinson’s disease (PD) and non-PD groups

Parameter	General population (non-PD cohort) (n = 55,664)	PD cohort (n = 10,104)	*P* value
Deaths, n	9167	2031	<0.001
PY per 1000 (95% CI)	183.09	36.23	
Rate per 1000 PY (95% CI)	50.07 (49.05 to 51.10)	56.06 (53.68 to 58.56)	
Unadjusted mortality rate ratio	1 (Reference)	1.12 (1.07 to 1.17)	<0.001
Age, gender, calendar year, social deprivation, smoking adjusted mortality rate ratio (95% CI)	1 (Reference)	1.14 (1.09 to 1.20)	<0.001

Abbreviations: PD, Parkinson’s disease; PY, person-years; CI, confidence interval.

**Table 3:** Adjusted mortality rates by age group, gender calendar year, social deprivation, and smoking

Variable	PD cohort				Non-PD cohort						
	Events	Person-years (1000)	^a^Adjusted mortality rate (95% CI)	*P* value	Events	Person-years (1000)	^a^Adjusted mortality rate (95% CI)	*P* value	Mortality rate ratio	^b^*P* value	^c^*P* value
Age group, y											
50–59	14	2.09	6.98 (3.0 to 10.97)	<0.001	51	10.63	4.46 (3.30 to 5.62)	<0.001	1.48 (0.80 to 2.76)	0.212	0.474
60–69	107	7.74	14.20 (11.30 to 17.09)		521	38.41	13.04 (11.81 to 14.27)		1.08 (0.87 to 1.33)	0.497	
70–79	571	14.95	39.57 (35.98 to 43.16)		2537	74.70	33.53 (31.90 to 35.16)		1.17 (1.07 to 1.28)	0.001	
80–89	1052	10.34	105.75 (98.78 to 112.71)		4813	53.35	92.91 (89.23 to 96.59)		1.14 (1.07 to 1.22)	<0.001	
>90	287	1.09	285.69 (250.05 to 321.32)		1245	5.97	223.98 (209.40 to 238.55)		1.28 (1.12 to 1.45)	<0.001	
Gender											
Male	1288	21.65	65.99 (61.77 to 70.20)	<0.001	5792	109.53	54.76 (52.41 to 57.12)	<0.001	1.18 (1.11 to 1.25)	<0.001	0.452
Female	743	14.58	51.82 (47.48 to 56.17)		3375	73.39	44.71 (42.67 to 46.74)		1.14 (1.06 to 1.23)	0.001	
Townsend quintile											
1 (least deprived)	510	10.37	55.65 (50.21 to 61.09)	0.0197	1939	47.19	44.25 (41.53 to 46.98)	<0.001	1.24 (1.13 to 1.37)	<0.001	0.323
2	482	8.54	58.85 (53.02 to 64.68)		2023	41.57	48.77 (45.90 to 51.64)		1.19 (1.08 to 1.32)	0.001	
3	404	6.69	66.03 (59.30 to 72.75)		1919	35.70	53.56 (50.29 to 56.83)		1.19 (1.08 to 1.32)	0.001	
4	291	5.05	61.08 (53.75 to 68.40)		1630	27.52	56.51 (52.48 to 60.54)		1.04 (0.92 to 1.18)	0.497	
5 (most deprived)	210	3.0	67.88 (57.89 to 77.87)		1049	17.67	59.36 (54.55 to 64.17)		1.09 (0.94 to 1.26)	0.246	
Missing data	134				607						
Smoking status											
Non-smoker	1035	20.40	53.87 (50.24 to 57.50)	<0.001	3649	82.57	44.21 (42.10 to 46.32)	<0.001	1.18 (1.11 to 1.26)	<0.001	0.086
Ex-smoker	716	10.76	65.14 (59.85 to 70.43)		3450	60.03	54.02 (51.62 to 56.43)		1.19 (1.10 to 1.28)	<0.001	
Current smoker	149	2.50	74.24 (61.70 to 86.78)		1378	23.88	74.74 (69.82 to 79.66)		0.96 (0.80 to 1.13)	0.604	
Missing data	131				690						

Abbreviations: PD, Parkinson’s disease; CI, confidence interval, y, years.

a Adjusted for age, gender calendar year, social deprivation, and smoking.

b Wald test for categorical variable.

c Wald test for multiplicative interaction.

## Data Availability

The authors have obtained the data for this study from IQVIA through a research license and do not own the dataset used and do not have permission to share the data. Access to THIN can be obtained through IQVIA by applying for a research license. More information on the availability of THIN data is available in the following URL https://www.iqvia.com/locations/uk-and-ireland/thin and permissions for data access can be obtained through https://www.iqvia.com/contact/general. The authors accessed the data in the same manner and had no special privileges to the data.
