# Supplementary material for: Mortality of People with Parkinson’s Disease in a Large UK-Based Cohort Study: Time Trends and Relationship to Disease Duration
Source: Mov Disord. Author manuscript; Available in PMC 2022 Jun 28. (PMC7612920; doi:10.1002/mds.28727)
Supplement: Supplementary information [file EMS146183-supplement-Supplementary_information.docx]

**Supplementary materials**

Database (The Health Improvement Network-THIN) with records of over 12 million people representative of UK population.

Between 2006 and 2016, all individuals 50 years and more.

n = 3,195,391

Excluded people in a practice with less than 6 months of follow-up

n = 110,005

(PD=3,756)

Potential study population (individuals 50 years and more) after exclusion.

n = 3,085,386

Excluded:

-all people with a history of PD prior to start of study n = 13,655.

-all those with RLS n = 31

Potential study population (individuals 50 years and more) after exclusion

n = 3,071,700

Individuals 50 years and more without incident PD diagnosis using Read code for

Parkinson’s disease and at

least 2 prescriptions of antiparkinsonian medications between 2006 and 2016.

n = 3,061,596

Total number with first recording of PD diagnosis using Read code for Parkinson’s disease and at least 2 prescriptions of antiparkinsonian medications between 2006 and 2016. These were included in the study (incident cases).

n = 10,104

PD-Parkinson’s disease. RLS-Restless Leg Syndrome.

Supplemental Figure 1. Flow chart showing identification of incident cases of PD between 2006 and 2016 in THIN using PD diagnosis Read code and two prescriptions of antiparkinsonian medication.

Supplemental Table 1: Unadjusted mortality rates in the PD and non-PD groups

|  | **PD-cohort** |  |  | **Non-PD cohort** |  |  |
| --- | --- | --- | --- | --- | --- | --- |
|  | Events | Person-Years (1000) | Mortality rate (95% CI) | Events | Person-Years | Mortality rate (95% CI) |
| **Overall** | 2,031 | 36.23 | 56.06 (53.68 to 58.56) | 9,167 | 183.09 | 50.07 (49.05 to 51.10) |
| **Age group** |  |  |  |  |  |  |
| 50 to 59 | 14 | 2.09 | 6.70 (3.97 to 11.31) | 51 | 10.63 | 4.80 (3.65 to 6.32) |
| 60 to 69 | 107 | 7.74 | 13.82 (11.43 to 16 70) | 521 | 38.41 | 13.56 (12.45 to 14.78) |
| 70 to 79 | 571 | 14.95 | 38.19 (35.18 to 41.45) | 2,537 | 74.70 | 33.96 (32.67 to 35.31) |
| 80 t0 89 | 1,052 | 10.34 | 101.67 (95.71 to 108.0) | 4,813 | 53.35 | 90.21 (87.70 to 92.80) |
| >90 | 287 | 1.09 | 263.42 (234.59 to 295.79) | 1,245 | 5.97 | 206.77 (195.55 to 218.63) |
| **Gender** |  |  |  |  |  |  |
| Male | 1,288 | 21.65 | 59.50 (56.34 to 62.84) | 5,792 | 109.53 | 52.80 (51.46 to 54.18) |
| Female | 743 | 14.58 | 50.96 (47.43 to 54.76) | 3,375 | 73.39 | 46.0 (44.46 to 47.56) |
| **Year** |  |  |  |  |  |  |
| 2007 | 22 | 0.43 | 51.47 (33.89 to 78.16) | 112 | 1.96 | 57.28 (47.60 to 68.93) |
| 2008 | 92 | 1.76 | 52.27 (42.62 to 64.14) | 512 | 7.84 | 62.28 (59.86 to 71.18) |
| 2009 | 135 | 3.01 | 44.86 (37.90 to 53.11) | 775 | 13.14 | 58.96 (54.94 to 63.26) |
| 2010 | 208 | 3.59 | 57.87 (50.52 to 66.30) | 906 | 15.96 | 56.77 (53.19 to 60.58) |
| 2011 | 209 | 4.09 | 51.08 (44.61 to 58.50) | 951 | 19.13 | 49.71 (46.65 to 52.97) |
| 2012 | 267 | 4.49 | 59.44 (52.72 to 67.02) | 1167 | 22.21 | 52.53 (49.60 to 55.63) |
| 2013 | 275 | 4.66 | 59.00 (52.43 to 66.41) | 1171 | 24.27 | 48.26 (45.57 to 51.10) |
| 2014 | 286 | 4.65 | 61.45 (54.72 to 69.00) | 1133 | 25.61 | 44.24 (41.74 to 46.89) |
| 2015 | 273 | 4.14 | 65.87 (58.50 to 74.17) | 1007 | 23.49 | 42.88 (40.31 to 45.61) |
| 2016 | 210 | 3.49 | 60.10 (52.50 to 68.81) | 703 | 20.58 | 34.16 (31.72 to 36.78) |
| **Townsend quintile** |  |  |  |  |  |  |
| 1 | 510 | 10.37 | 49.18 (45.10 to 53.64) | 1,939 | 47.19 | 41.09 (39.30 to 42.96) |
| 2 | 482 | 8.54 | 56.44 (51.62 to 61.71) | 2,023 | 41.57 | 48.67 (46.59 to 50.83) |
| 3 | 404 | 6.69 | 60.43 (54.81 to 66.62) | 1,919 | 35.70 | 53.76 (51.40 to 56.21) |
| 4 | 291 | 5.05 | 57.68 (51.42 to 64.70) | 1,630 | 27.52 | 59.23 (56.42 to 62.18) |
| 5 | 210 | 3.0 | 70.28 (61.39 to 80.46) | 1,049 | 17.67 | 59.38 (55.89 to 63.08) |
| No records | 134 |  |  | 607 |  |  |
| **Smoking status** |  |  |  |  |  |  |
| Non-smoker | 1035 | 20.40 | 50.74 (47.74 to 53.92) | 3649 | 82.57 | 44.19 (42.78 to 45.65) |
| Ex-smoker | 716 | 10.76 | 66.52 (61.83 to 71.58) | 3450 | 60.03 | 60.03 (55.58 to 59.42) |
| Current smoker | 149 | 2.50 | 59.55 (50.72 to 69.92) | 1378 | 23.88 | 23.88 (54.74 to 60.84) |
| No records | 131 |  |  | 690 |  |  |
| **Year(s) after PD diagnosis** | | |  |  |  |  |
| First year | 329 | 9.44 | 34.84 (31.27 to 38.81) | 2959 | 48.83 | 60.60 (58.45 to 62.82) |
| Second year | 362 | 7.64 | 47.40 (42.76 to 52.54) | 1,926 | 37.73 | 51.05 (48.82 to 53.38) |
| Third year | 354 | 5.95 | 59.49 (53.61 to 66.03) | 1,343 | 29.10 | 46.16 (43.75 to 48.69) |
| Fourth year | 298 | 4.48 | 66.54 (59.39 to 74.54) | 943 | 21.9 | 43.06 (40.40 to 45.90) |
| Fifth year | 238 | 3.25 | 73.16 (64.43 to 83.07) | 741 | 16.23 | 45.66 (42.49 to 49.07) |
| Sixth year | 180 | 2.29 | 78.74 (68.04 to 91.13) | 519 | 11.56 | 44.90 (41.20 to 48.93) |
| Seventh year | 134 | 1.49 | 89.84 (75.85 to 106.41) | 314 | 7.83 | 40.11 (35.91 to 44.80) |
| Eighth year | 73 | 0.91 | 80.54 (64.03 to 101.31) | 199 | 5.06 | 39.35 (34.25 to 45.22) |
| Ninth year | 38 | 0.49 | 76.91 (55.96 to 105.70) | 147 | 3.01 | 48.87 (41.57 to 57.44) |
| Tenth year | 20 | 0.22 | 89.05 (57.45 to 138.02) | 54 | 1.44 | 37.51 (28.73 to 48.97) |
|  |  |  |  |  |  |  |

PD-Parkinson’s disease

Supplemental table 2: Adjusted mortality rates (adjusted for age, gender, calendar year, social deprivation and smoking) following PD diagnosis/index date for non-PD group

| **Variables** | **PD cohort** | | |  | **Non-PD cohort** | | | | | |  |
| --- | --- | --- | --- | --- | --- | --- | --- | --- | --- | --- | --- |
|  | Events | Person-Years (1000) | *Adjusted Mortality rate (95% CI) | p-value | Events | Person-Years  (1000) | *Adjusted Mortality rate (95% CI) | p-value | Mortality rate ratio | **p-value | ***p-value |
| **Years following diagnosis/index date** | | | | | | | | | | | |
| First year | 362 | 7.64 | 43.46 (38.43 to 48.48) | <0.001 | 1,926 | 37.73 | 59.62 (56.34 to 62.90) | <0.001 | 0.70 (0.63 to 0.78) | <0.001 | 0.001 |
| Second year | 354 | 5.95 | 50.56 (44.88 to 56.24) |  | 1,343 | 29.10 | 51.35 (48.46 to 54.23) |  | 0.96 (0.86 to 1.07) | 0.499 |  |
| Third year | 298 | 4.48 | 66.13 (58.68 to 73.58) |  | 943 | 21.9 | 46.20 (43.31 to 49.09) |  | 1.42 (1.26 to 1.60) | <0.001 |  |
| Fourth year | 238 | 3.25 | 74.48 (66.02 to 82.93) |  | 741 | 16.23 | 43.06 (39.78 to 46.35) |  | 1.74 (1.54 to 1.98) | <0.001 |  |
| Fifth year | 180 | 2.29 | 74.66 (64.46 to 84.87) |  | 519 | 11.56 | 41.69 (38.27 to 45.11) |  | 1.84 (1.59 to 2.12) | <0.001 |  |
| Sixth year | 134 | 1.49 | 88.69 (74.43 to 102.95) |  | 314 | 7.83 | 40.62 (36.54 to 44.69) |  | 2.27 (1.92 to 2.71) | <0.001 |  |
| Seventh year | 73 | 0.91 | 87.58 (70.32 to 104.84) |  | 199 | 5.06 | 37.13 (32.44 to 41.83) |  | 2.51 (2.01 to 3.13) | <0.001 |  |
| Eighth year | 38 | 0.49 | 73.21 (52.41 to 94.00) |  | 147 | 3.01 | 42.06 (35.72 to 48.41) |  | 1.89 (1.37 to 2.59) | <0.001 |  |
| Ninth year | 20 | 0.22 | 94.00 (58.84 to 129.07) |  | 54 | 1.44 | 32.01 (24.43 to 39.59) |  | 3.23 (2.11 to 4.94) | <0.001 |  |
| Tenth year | 5 | 0.06 | 94.26 (9.79 to 178.74) |  | 22 | 0.41 | 46.05 (28.06 to 64.03) |  | 2.26 (0.82 to 6.24) | 0.100 |  |

PD-Parkinson’s disease. *Adjusted for age, gender, calendar year, social deprivation and smoking. **Wald test for categorical variable. ***Wald test for multiplicative interaction.

Supplemental Figure 2. Mortality rate over time adjusted for age, gender,

time since diagnosis/index date, by follow-up year since diagnosis/entry.

(a)

(b)

Supplemental Figure 3. Sensitivity analysis showing (a) mortality rates following PD diagnosis/drug prescription/index date adjusted for age, sex, calendar year and social deprivation; (b) mortality rates following PD diagnosis/ drug prescription/index date adjusted for age, sex, calendar year, social deprivation and smoking.

Supplemental Figure 4. Association between age and mortality by PD showing mortality rates (three-knot cubic spine transformation) adjusted for sex, calendar year, social deprivation and smoking.

Supplemental table 3: Demographic characteristics of people with PD using different diagnostic definitions.

| **Variables** | **PD diagnosis Read code** | **PD diagnosis Read code plus 1 drug prescription** | **PD diagnosis Read code plus 2 drug prescriptions**  **(prior to study follow-up)** | **PD diagnosis Read code plus 2 drug prescriptions** |
| --- | --- | --- | --- | --- |
| **Gender *n (%)*** |  |  |  |  |
| Men (%) | 173(56.9) | 792(59.7) | 2,054(54.7) | 6,135(60.7) |
| Women (%) | 117(43.1) | 600(40.3) | 1,702(45.3) | 3,969(39.3) |
| **Age group *n (%)*** |  |  |  |  |
| 50 to 59 | 78(5.7) | 18(6.21) | 327(8.9) | 790(7.82) |
| 60 to 69 | 282(20.5) | 46(15.9) | 856(23.3) | 2,328(23.0) |
| 70 to 79 | 507(36.8) | 104(35.9) | 1,387(37.7) | 4,187(41.5) |
| 80 to 89 | 442(32.2) | 106(36.6) | 999(27.1) | 2,554(25.3) |
| 90 and over | 66(4.80) | 16(5.5) | 113(3.1) | 242(2.4) |
| **Townsend quintile** |  |  |  |  |
| 1(least deprived) | 331(23.8) | 87(30.0) | 838(22.3) | 2,811(27.8) |
| 2 | 325(23.4) | 69(23.8) | 895(23.8) | 2.320(23.0) |
| 3 | 257(18.5) | 41(14.1) | 673(17.9) | 1,901(18.8) |
| 4 | 225(16.2) | 41(14.1) | 583(15.5) | 1,441(14.3) |
| 5(most deprived) | 136(9.77) | 36(12.4) | 355(9.5) | 895(8.7) |
| Not recorded | 118(8.5) | 16(5.5) | 412(10.9) | 736(7.3) |

PD-Parkinson’s disease
